# Optimization of Serum and Salivary Cortisol Interpolation for Time-Dependent Modeling Frameworks in Healthy Adult Males

**DOI:** 10.3390/sports13040112

**Published:** 2025-04-09

**Authors:** Nathaniel T. Berry, Travis Anderson, Christopher K. Rhea, Laurie Wideman

**Affiliations:** 1 Department of Kinesiology, University of North Carolina at Greensboro, Greensboro, NC 27412, USA; t_ander2@uncg.edu; 2 Ellmer College of Health Sciences, Old Dominion University, Norfolk, VA 23529, USA; crhea@odu.edu

**Keywords:** cortisol, interpolation, optimization

## Abstract

Cortisol is an important marker of hypothalamic-pituitary-adrenal function and follows robust circadian and diurnal rhythms. However, biomarker sampling protocols can be labor-intensive and cost-prohibitive. Objectives: Explore analytical approaches that can handle differing biological sampling frequencies to maximize these data in more detailed and time-dependent analyses. Methods: Healthy adult males [N = 8; 26.1 (±3.1) years; 176.4 (±8.6) cm; 73.1 (±12.0) kg)] completed two 24 h admissions: one at rest and one including a high-intensity exercise session on the cycle ergometer. Serum and salivary cortisol were sampled every 60 and 120 min, respectively. Six alternative sampling profiles were defined by downsampling from the observed data and creating two intermittent sampling profiles. A polynomial (1–6 degrees) validation process was performed, and interpolation was conducted to match the observed data. Model fit and performance were assessed using the coefficient of determination (R^2^) and the root mean square error (RMSE), as well as an examination of the equivalence, via two one-sided t-tests (TOST), of 24 h cortisol output between the observed and interpolated data. Results: Mean serum cortisol output was higher than salivary cortisol (*p* < 0.001), and no effect was observed for condition (*p* = 0.61). Second- and third-degree polynomial regressions were determined to be the optimal models for fitting salivary. TOST tests determined that serum data and estimated 24 h output from these models (with interpolation) provided statistically similar estimates to the observed data (*p* < 0.05). Conclusions: Second- and third-degree polynomial fits of salivary and serum cortisol provide a reasonable means for interpolation without introducing bias into estimates of 24 h output. This allows researchers to sample biomarkers at biologically relevant frequencies and subsequently match necessary sampling frequencies during the data processing stage of various machine learning workflows.

## 1. Introduction

Cortisol is a steroid-derived lipophilic hormone primarily found in circulation and can be measured in various biological fluids, including serum, saliva, and urine. An important marker of hypothalamic–pituitary–adrenal function, cortisol follows robust circadian and diurnal rhythms. These circadian rhythms are proposed as one of the primary regulators of the peripheral biological clocks [1,2]; consequently, disruption of cortisol regulation is associated with a plethora of disorders [1,3]. In addition, serum cortisol is considered a significant diagnostic marker of cardiovascular and metabolic disease [1,2]. Disruption in the circadian rhythm of serum cortisol occurs with aging and in response to shift work and other sleep disturbances, but the 24 h profile is less impacted by acute changes in environmental and/or behavioral conditions [1]. For example, clear increases in cortisol are observed after carbohydrate-rich meals, but the overall shape of the circadian profile is unaltered [4].

Approximately 80% of circulating cortisol is tightly bound to corticotropin-binding globulin in a 1:1 ratio [5], while approximately 10% is loosely bound to albumin. The remaining ~10% is considered the free portion and can diffuse into target cells. This free portion can also passively diffuse across salivary gland membranes and, thus, is detectable in saliva. Salivary cortisol assessment is becoming increasingly popular due to the noninvasive nature of collection and relative stability of the sample at room temperature or in commercially available refrigerators and freezers [6]. As a result, participants can collect samples outside of the laboratory environment, improving ecological validity. In addition, salivary cortisol is commonly preferred for assessing diurnal slopes, which have been associated with a myriad of psychophysiological conditions [7]. Although there are distinct advantages to diurnal slope measures (e.g., a limited number of samples required), and this approach has provided valuable findings across scientific disciplines [7,8,9,10,11,12], some disordered or abnormal cortisol profiles may only be detectable with greater resolution of the circadian profile.

A fundamental first step in biological sampling is to determine the appropriate sampling interval from both a modeling and physiological perspective [13,14]. Depending on the design and methods chosen, these sampling intervals may occur at equidistant or non-equidistant sampling frequencies. The choice in sampling method should occur at a set frequency or at specific points in time that represent significant changes in concentration—considering the research design and half-life of the biomarker of interest [13]. Technological advances have made the assessment of invasive and noninvasive biomarkers increasingly accessible [15]. As a result, researchers have access to previously unavailable (cumbersome to collect, at a minimum) data.

Coupled with increasingly integrated research designs, additional and more sophisticated analytical approaches are necessary to answer more complex research questions. Some of these approaches require more frequent sampling and/or an equal number of observations between all markers being assessed. Although technology has made this easier, meeting these requirements can be both time- and cost-prohibitive. For example, a ~16–19 min half-life for growth hormone [14] compared to a ~66 min half-life for cortisol [16] means capturing important changes in each respective hormone requires drastically different sampling intervals. In this instance, many critical changes in growth hormone would be missed by sampling every hour, whereas sampling every 10 min would be unnecessary and a misallocation of resources to capture the circadian pattern of cortisol.

The overall objective of these analyses is to explore analytical approaches that can help researchers address issues of necessarily nonconcurrent biological sampling frequencies, so that these data can be leveraged in more complex and time-dependent analyses. More specifically, these methods apply to situations where multiple biomarkers are being assessed, and each biomarker has a distinctly different half-life, or responsiveness to external perturbations (e.g., exercise, diet, sleep). If planned analytical methods require equal data length, such characteristics may result in unnecessary sampling of more stable biomarkers to match that of more responsive measures. Establishing the minimum number of samples and the optimal number of model parameters necessary to capture the trends in serum and salivary cortisol throughout the 24 h profile is germane to clinical, research, and applied settings.

The purpose of this study was to take a data-driven approach to examine the relations between various sampling methodologies and regression models to fit these data on the estimation of circadian and diurnal trends in serum and salivary cortisol and the ability of various models to estimate 24 h serum and salivary cortisol output. We hypothesized that we would be able to determine an optimal regression model to fit serum and salivary cortisol and that we would be able to interpolate along these models to reliably estimate 24 h cortisol output. The data-driven approach guarantees decisions are objective and evidence-based, guided by data analysis and interpretation. As such, we did not assume that any model would reflect our ground truth data and minimized potential bias in our analyses by eliminating *a priori* hypotheses about which model would perform best.

## 2. Materials and Methods

*Overview*. This study included a within-subjects randomized block design involving intensive serial measurements of blood and saliva taken throughout a 24 h period. Adult males were recruited for this study and reported to the laboratory on two occasions. Each visit consisted of a 24 h admission where subjects performed minimal activity (rest) or a single high-intensity exercise bout; conditions were randomly assigned and separated by a minimum of 8 weeks to allow for recovery of blood volume. Serum cortisol was assessed every hour and salivary cortisol was collected every 2 h during each admission. Methodological details related to other aspects of this study have been published previously [17,18].

*Sample*. Adult males, ages 18–35 years, who regularly participated in moderate to vigorous exercise were recruited from the University and surrounding areas to participate in this study. Exercise history was confirmed as part of a standard medical history questionnaire, and any individual not meeting the predetermined criteria was excluded from the study. All individuals were free of any known metabolic, cardiovascular, or pulmonary disease and had <18% body fat (assessed with COSMED’s BOD POD). Anyone with acute or chronic health conditions or taking medications for cardiovascular or metabolic disease, mental health conditions, endocrine disorders, or any other infectious condition was excluded from this study.

*The 24 h Visit*. Participants reported to the laboratory at 05:30 for the 24 h admission that began at 06:00. Upon arrival, an intravenous catheter was placed in either the radial or antecubital vein. Whole blood was drawn each hour, beginning at 06:00 and continuing every hour until 06:00 the following morning (total number of samples per admission = 25). Salivary samples were collected, beginning at 06:00 and continuing every two hours until 06:00 the following morning (total number of samples per admission = 13). Individuals ate breakfast at approximately 07:30 and were restricted to water between 08:00 and 10:00 to standardize macronutrient intake before and during the exercise bout (at 10:00); restriction of dietary intake was consistent during both conditions. Individuals ate lunch at approximately 13:00 and dinner at approximately 20:00. All food and beverages consumed by the participants were detailed in a dietary log, and participants were asked to consume foods of similar macronutrient composition during the second profile visit. Similarly, subjects were asked to maintain a similar nighttime routine during each visit; participants were permitted to go to bed at their discretion, with a mandatory lights-out policy at 23:00.

*Exercise Protocol*. Following a 5 min warmup at a self-selected workload (≤50 watts), participants began the high-intensity exercise session consisting of five 30 s maximal efforts on a Lode Excaliber Sport cycle ergometer. The force applied to the flywheel was defined as F = M × 7.5%, where M is body mass in kg. Each of the five 30 s bouts was separated by a 3 min active recovery period on the cycle ergometer. Following the final bout, participants completed a 3–5 min cool down at a self-selected load (≤50 watts).

*Biological Sample Collection and Analysis*. The intravenous catheter was connected to a normal saline drip with a keep-vein-open protocol to maintain line patency (20–30 mL/h). A total of 3 ml of whole blood was collected in a serum separator tube through a closed system. Participants were volume-repleted with the waste, and a ~3–5 mL bolus of normal saline after each sample was taken. Samples were allowed to clot for 20–40 min and then centrifuged for 12 min at 3000 g. Serum was aliquoted into 1.5 mL storage tubes and frozen at −80 °C until assayed. Salivary cortisol was collected in a sterile storage tube through passive drool and stored at −80 °C until assayed. Serum and salivary cortisol were assayed using commercially available enzyme-linked immunosorbent assays (R&D Systems, Minneapolis, MN, USA).

*Analytical Methods & Data Processing*. Serum sampled every hour (Q60) and saliva sampled every two hours (Q120) were the highest sampling frequencies collected for each specimen type. These data, and any model established from these data, are defined as the ground truth and the basis for which all subsequent comparisons are made. Alternative sampling methods were defined by down-sampling in 2 h increments from Q120 until a minimum sampling method of one sample every 6 h (Q360) was met. A minimum of Q360 was chosen to maintain the necessary degrees of freedom to fit a third-order polynomial. In addition, two non-equidistant sampling methodologies were examined (i.e., INT1 and INT2). Timing intervals for INT1 and INT2 were chosen to replicate a potential 24 h cortisol sampling methodology, with a total number of 6 and 5 samples, respectively. Many additional combinations of non-equidistant sampling methodologies could be chosen for analysis, but we have chosen these combinations of time points to replicate potential and convenient sampling windows for research or clinical application.

For each sampling method, regression across multiple polynomial degrees was fit to each profile on an individual-by-condition basis. Additional details on the sampling methods that were assessed can be found in Table 1. Maximum polynomial degrees were chosen based on the maximum number of available degrees of freedom. We purposefully began by underfitting each profile (linear regression) and incrementally increased model complexity to overfit these data to investigate the relations between sampling method, model complexity, and interpolation. We have previously examined how trends in serum and salivary cortisol, as well as the relation between serum and salivary cortisol, differ between rest and exercise conditions [19]. The present analysis is meant to focus specifically on a method for which group-wise data is not available and equal sampling methods are needed for subsequent modeling frameworks.

Three key steps and comparisons were made hereafter to determine the optimal parameters for interpolation from varying sampling frequencies. First, we fit multiple regression models (at varying degrees) to each dataset (i.e., ground truth and alternative sampling methods) and examined model fit with the coefficient of determination (R^2^). Importantly, these values do not consider model complexity and degrees of freedom. As part of this first step, we also estimated the 24 h total cortisol output for each sampling method to compare how sampling frequency impacts the 24 h cortisol estimate. Secondly, we compared the performance of each regression model fit to the alternative sampling methods relative to the ground truth data. Model fit (R^2^) and the root mean squared error (RMSE) were calculated to provide a measure of how well the model from each of the alternative sampling methods performed relative to the full, ground truth data. Lastly, we used the models from each of the alternative sampling methods to interpolate missing values, obtaining Q60 and Q120 profiles for serum and salivary cortisol, respectively. The error (RMSE) between the ground truth models and interpolated profiles was used to compare model performance between sampling methods and regression models. Upon consideration of the above data, we determined the optimum parameters for interpolation and compared the estimates of total serum and salivary cortisol output from select regression models to the estimated 24 h output from the ground truth data.

**Figure 1:**
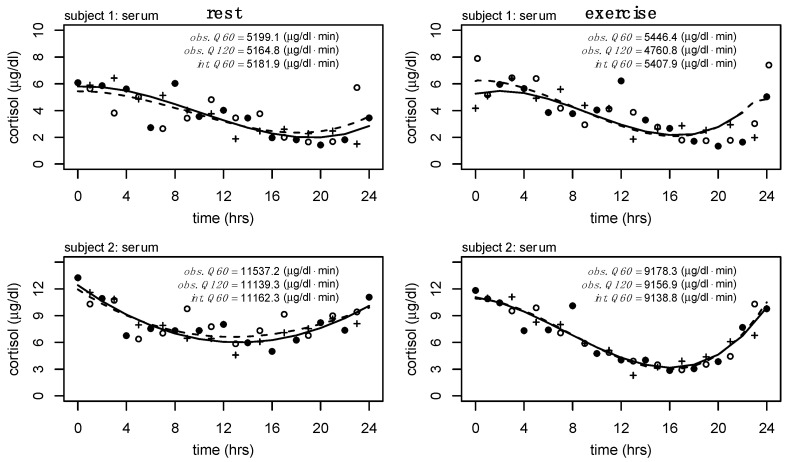
Observed, fitted, and interpolated serum cortisol data for two subjects during rest and exercise. • represents observed data at sampled every 120 min (Q120), while a combination of closed and open circles represents Q60 data; + represents interpolated values; — represents the model (degree 3 polynomial) fitted to the Q120 data (used for interpolation); -- represents model (degree 3 polynomial) fitted to the Q60 data (for comparison). Estimated 24 h serum cortisol output from Q60 (obs. Q60). Estimated 24 h serum cortisol output from Q120 (obs. Q120). Estimated 24 h serum cortisol from interpolated output (int. Q60).

**Figure 2:**
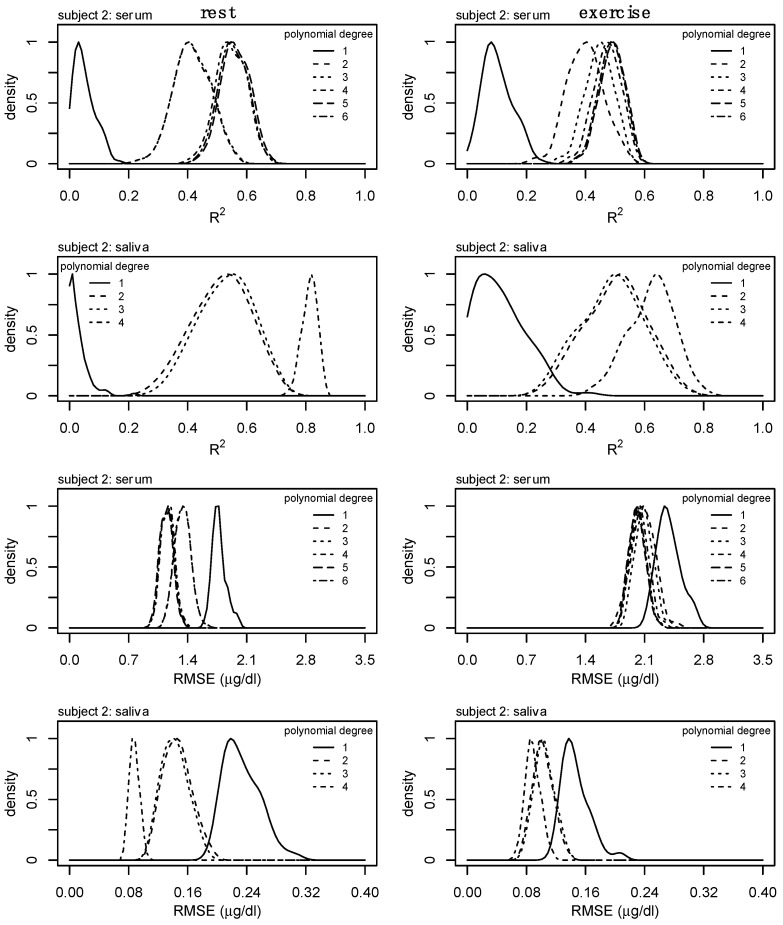
Kernel density estimations for R^2^ and RMSE values from the 100-iterations performed during the interpolation process. Distributions represent the model fit (R^2^) and RMSE from the interpolation performed following each regression model fit to the data sampled every 60 and 120 min (Q60 and Q120) profiles for serum and salivary cortisol, respectively. High variance is represented by wider distributions, while high bias between models is represented by drastic shifts in peaks. Regression models of polynomial degrees (2, 3) converged on similar measures of model performance. Data were presented from a single subject; this process was repeated for each alternative profile across all subjects independently.

Interpolation was performed for each alternative sampling method to obtain Q60 and Q120 values for serum and salivary cortisol, respectively. For example, the Q120 serum cortisol would contain an interpolated value every second value (i.e., every other hour). Error was added to each of the interpolated values on a model-by-model basis by generating Gaussian distributed values within the 95% confidence interval of the residuals. The process of adding random values to the interpolated process results in changes to model performance (e.g., R^2^ and RMSE) at each iteration. In consideration of this, the generation of random error was repeated 100 times for each sampling method to generate a distribution for both R^2^ and RMSE. The R^2^ and RMSE values corresponding to the peak density from each of these distributions were used for subsequent analyses. A representation of the interpolation process is provided in Figure 1, and an example of the distributions produced from the 100 iterations of interpolation with random error is provided in Figure 2. To regenerate the optimal model for each polynomial degree, a unique seed was generated at each iteration.

*Statistical Analysis*. Mean differences in 24 h serum and salivary cortisol at rest and exercise from the ground truth data (i.e., Q60 serum and Q120 salivary cortisol) were tested using an analysis of variance. Equivalence between estimated 24 h serum and salivary cortisol output following interpolation for select regression models with the observed Q60 (serum) or Q120 (saliva) estimates were performed using two one-sided tests (TOST). The region of similarity, ε, was set at 0.25. Significance was set at *p* < 0.05, and distributional normality was assessed prior to all analyses.

The *optimal* model was determined through careful analysis and comparison of the (1) average model fit (R^2^), (2) error (RMSE) of the regression models, and (3) estimated cortisol output. We utilized a data-driven approach to guarantee that these decisions were objective and evidence-based, guided by data analysis and interpretation. As such, no *a priori* hypotheses were made about which model would perform best. All processing and statistical procedures were performed using R statistics [20], available packages [21,22,23], and custom scripts.

## 3. Results

Subjects (N = 8) were 26.1 (±3.1) years of age with an average height of 176.4 (±8.6) cm and a body mass of 73.1 (±12.0) kg. We examined differences in 24 h serum and salivary cortisol output between rest and exercise conditions using the ground truth data (i.e., Q60 and Q120 data for serum and salivary cortisol, respectively).

**Figure 3:**
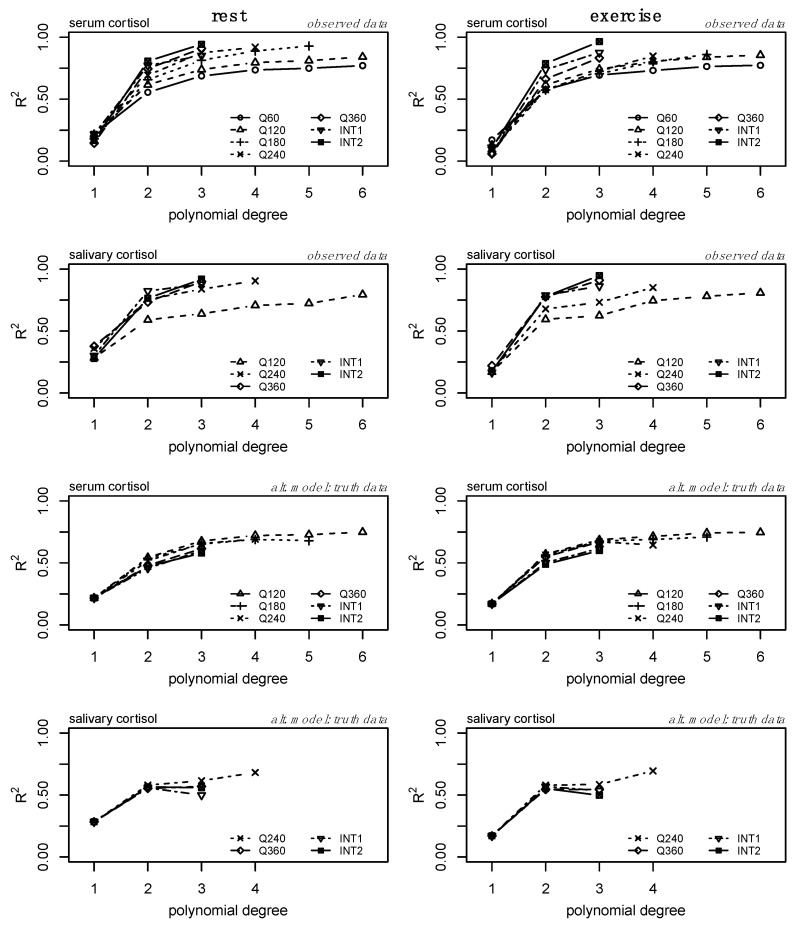
Coefficient of variation (R^2^) from each of the models fit to the observed (i.e., ground truth and alternative) data. Data from the observed models (top two rows) represent the fit of each model (polynomial degree) across each sampling frequency (e.g., Q60, Q120, etc.). Note that these values do not consider model complexity and available degrees of freedom. Models from each of the alternative sampling methods were fit to the ground truth data and model fit was compared (bottom two rows). These data highlight how models from various sampling frequencies perform relative to the highest sampling frequency available (e.g., Q60 and Q120 for serum and salivary cortisol, respectively). Data are presented as the means.

The average model fit (R^2^) for each model is provided in Figure 3. Model fit increased similarly across sampling methods and conditions for serum and salivary cortisol. For serum, the fit plateaued around degree 3, while the fit plateaued around degree 2 for salivary cortisol. Figure 3 should serve as a baseline for understanding and comparing subsequent analyses.

**Figure 4:**
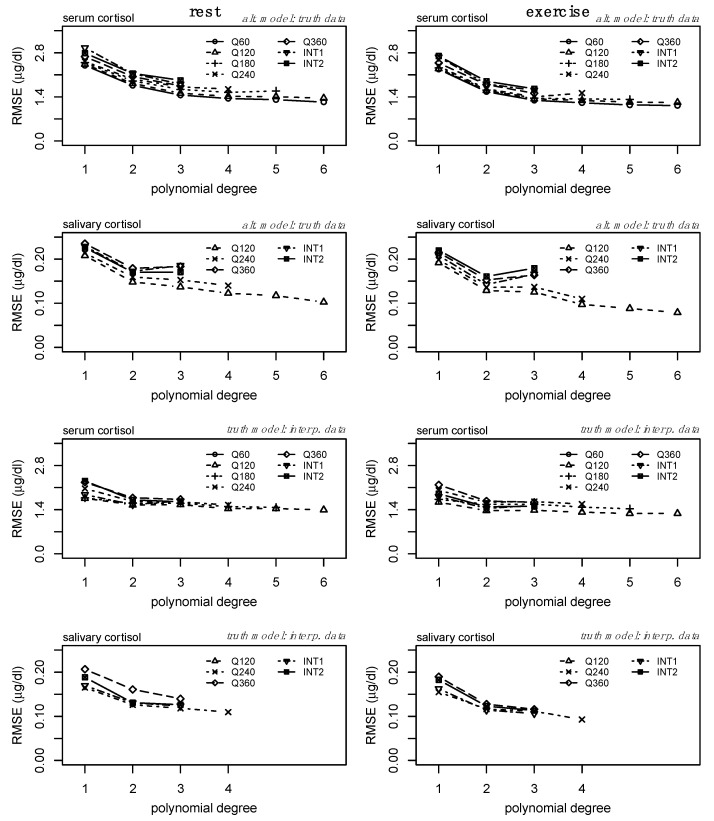
Root mean square error (RMSE) from the assessment of each alternative model compared to the ground truth data (top two rows) and the models generated from the ground truth data for each sampling method with interpolation (bottom two rows). Data in the top two rows correspond to the alternative models presented in Figure 3. Data are presented as means.

Secondly, we compared the performance of each regression model fit to the alternative sampling methods relative to the ground truth data (Figure 3, bottom two rows). Model fit (R^2^) increased similarly for each model defined from the alternative datasets, demonstrating the relationship between increasing polynomial degree across sampling methods and the ability to represent the ground truth data. However, there are clear shifts in model performance (particularly between models fit to lower sampling frequencies) in the bottom two rows of Figure 3, representing the effect of overfitting at higher orders and lower sampling frequencies. For serum, increases in R^2^ were minimal above polynomial degree 3 (Figure 3, third row), but the performance of models derived from lower sampling frequencies performed worse than those of higher sampling frequencies. Similar and more drastic shifts in model performance can be observed within the salivary cortisol data. Models derived from each sampling method appeared to perform similarly at degree 2, but a reduction in performance is observed (Figure 3, bottom row) at degree 3 for several sampling frequencies. The error (RMSE) between each of the alternative models and the ground truth data is provided in Figure 4 (top two rows) and follows an inverse pattern as presented in Figure 3 and described above.

Thirdly, we examined the error (i.e., RMSE) between the interpolated profiles and the ground truth models. We observe a convergence of RMSE across each of the alternative sampling methods at polynomial degree 3 (Figure 4, third row) for serum cortisol. Specifically for salivary cortisol, there are slight reductions in RMSE from linear models to second-degree polynomial regression models (Figure 4, bottom row).

To further test the clinical impact of the interpolation process using various alternative sampling methods, we tested equivalence between estimates of 24 h serum and salivary cortisol output for each of the interpolated profiles relative to the estimates of 24 h serum and salivary cortisol output established from the ground truth data (i.e., Q60 and Q120 data for serum and salivary cortisol, respectively). These data are presented in Table 2. We observed statistical similarity between these profiles in all cases, suggesting agreement in the total output estimations following interpolation with the observed estimates of serum and salivary cortisol output, respectively. Confidence intervals from the two one-sided t-tests are provided in Table 3.

Consideration of the R^2^ and RMSE of the models from the alternative sampling methods relative to the ground truth data, as well as the RMSE of the interpolated profiles compared to the ground truth data, suggests the third-degree polynomial as the optimal model to fit individual-level serum cortisol profiles across a variety of sampling methods. Specific to salivary cortisol, we observe slight reductions in RMSE from linear models to second-degree polynomial regression models (Figure 4, bottom row). Considering the drastic increases in model fit (Figure 3, bottom row) and reduction in RMSE from the linear model to second-degree polynomial of the alternate model (Figure 4, second row), and the potential impact of the random error added to the interpolation process, we consider the second-degree polynomial to be the optimal model to fit individual level salivary cortisol data across a variety of sampling methods. Less drastic reductions in error between linear regression and regression models of degree 2 may be the result of less complex trends within the raw salivary cortisol profiles. Furthermore, estimates of 24 h serum and salivary cortisol for each of these sampling methods and interpolation from degree 3 and degree 2 for serum and salivary cortisol, respectively, were determined equivalent to the ground truth data. Figure 2 further supports these findings, providing a representation of the interpolation process as well as the variance and bias produced by each model. High variance is represented by wider distributions, while high bias between models is represented by drastic shifts in peaks. Although these data (Figure 2) represent a single individual, there is a clear convergence between models of polynomial degree (2, 3) for serum and salivary cortisol profiles. 

**Figure 5:**
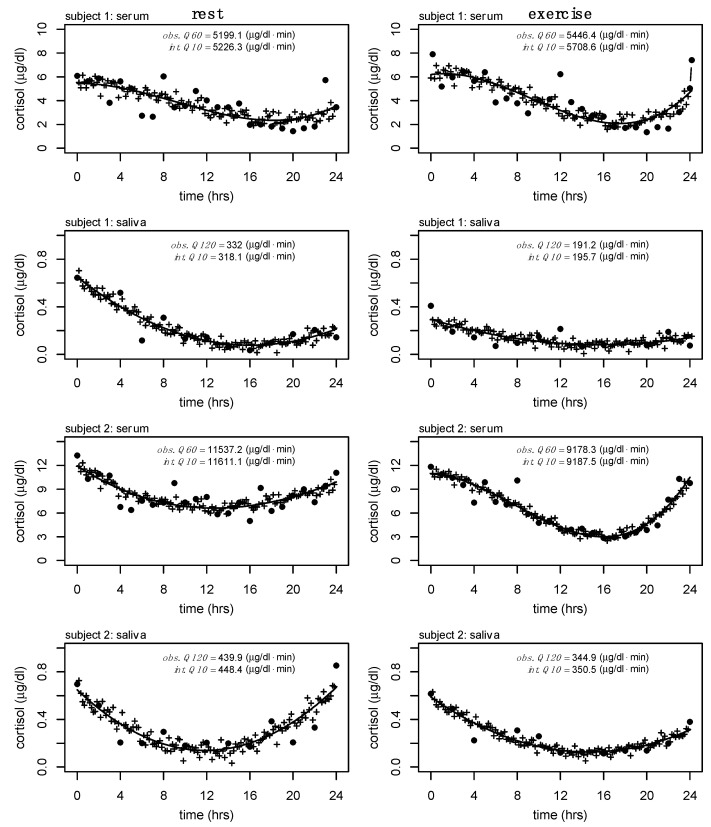
Observed, fitted, and interpolated serum and salivary cortisol data for two subjects during rest and exercise. • represents observed data sampled every 60 min (Q60, serum) and 120 min (Q120, saliva); — represents the model fit to the observed data; + represents interpolated values (Q10). Estimated 24 h serum and salivary cortisol output from Q60 (obs. Q60). Estimated 24 h serum and salivary cortisol output from Q120 (obs. Q120). Estimated 24 h serum and salivary cortisol from interpolated output (int. Q60). Polynomial regressions of degree 3 (serum) and degree 2 (saliva) were used to fit the serum (Q60) and salivary (Q120) cortisol data, respectively.

A final exploratory analysis where Q60 serum cortisol and Q120 salivary cortisol were interpolated to Q10 was performed to show the feasibility of these methods outside of the available data presented here (see Figure 5). This analysis is relevant to future work where other biomarkers are sampled at a higher frequency (e.g., growth hormone, frequently assessed at Q10).

## 4. Discussion

This study examined the relationships between various sampling methodologies and methods of interpolation for 24 h serum and salivary cortisol output to establish an analytical approach to help researchers address issues of necessarily nonconcurrent biological sampling frequencies, as well as a method of processing these profiles to make them more easily accessible in other analytical frameworks. We took a data-driven approach to examine these relationships and establish the minimal number of samples and the optimal number of model parameters necessary to capture these trends. From these findings, we show that higher serial sampling frequencies reduce the overall bias of 24 h serum and salivary cortisol but that second- and third-degree polynomial fits of salivary and serum cortisol provide a reasonable means for interpolation and that these interpolated values, across serial and intermittent sampling methods, provide a method of adequately estimating 24 h output.

A polynomial validation process, ranging from degree 1 up to degree 6, was performed on the ground truth and each alternative sampling methodology to establish the baseline performance of these models. Subsequent measures of fit (i.e., R^2^) and error (i.e., RMSE) between each alternative model and the ground truth data provided an idea of how well each of the models fit to the alternative profiles that represented the ground truth data. Our data-driven approach suggests that second- and third-degree polynomial fits for salivary and serum cortisol provide reasonable interpolation without biasing estimates of 24 h cortisol output, while avoiding overfitting. This allows researchers to sample biomarkers at biologically relevant frequencies and subsequently match necessary sampling frequencies during the data processing stage of various machine learning workflows. While the present methods focused on serum and salivary cortisol, the analytic approach could be applied to work with other biomarkers.

Cortisol is an important marker of hypothalamic-pituitary-adrenal function with robust circadian and diurnal rhythms. Twenty-four-hour hormone profiles can help elucidate changes to these rhythms due to acute and chronic adaptations and have long been of interest to clinicians and researchers. In the previously discussed comparative example of growth hormone and cortisol, where each biomarker has drastically different half-lives and secretory profiles, it likely does not make sense to measure each biomarker at equal frequencies. While both biomarkers share regulatory pathways, and each follows circadian patterns of regulation, the pulsatile nature of growth hormone requires significantly higher sampling frequencies (half-life ~16–19 min) to capture important changes in total output compared to cortisol. Growth hormone is often sampled at Q10 and though we do not have cortisol samples at this frequency, we have provided a theoretical representation of the interpolation process from serum (Q60) and salivary (Q120) cortisol to what Q10 would look like in our analyses (see Figure 5). Comparative examples of serially sampled 24 h cortisol output, at varying frequencies, can be observed elsewhere [24,25,26].

The potential utility of salivary cortisol in multiple situations is exciting due to its ease of collection and less strict processing and storing requirements compared to serum cortisol [6]. Salivary cortisol has been demonstrated to be highly correlated with serum cortisol at rest [27] and following physiological perturbations [28,29] and has been recommended as a potential surrogate for serum cortisol in deconvolution [30]. Despite these findings, it is important to note that saliva is a unique biological fluid that has considerations distinct from the assessment of cortisol in serum. Cortisol is a relatively small hormone (362.46 Da), and thus, the free portion readily diffuses across the membrane and is saliva flow-rate independent [31,32]. However, salivary glands have a high concentration of the enzyme hydroxysteroid dehydrogenase-2 [33,34], which rapidly converts cortisol to the less active cortisone. Moreover, binding protein dynamics, including the thermocouple between CBG and cortisol [35], add additional sources of variance when using salivary cortisol as a proxy for total cortisol in circulation.

Previous work has examined the individual and coupled changes in serum and salivary cortisol throughout the course of the 24 h period [19]. Here, mixed-effects models were used to examine the changes in serum and salivary cortisol throughout the course of the 24 h period and results indicated differing degrees of model complexity for serum versus salivary cortisol. Intuitively, this work [19] suggests that third-degree polynomials and second-degree polynomials adequately represented trends in serum and salivary cortisol throughout the 24 h period, aligning with the results and supporting the conclusions made herein.

The analytic methods explored here may apply in instances where biomarkers and other wearable device data are collected simultaneously. Rapid technological advancements in sensor systems increase the availability of serial measurements across extended periods of time in medicine and sport. These advancements provide an opportunity for new and innovative solutions to help decipher unique problems related to analytical methods—specifically those related to time series and the dynamics of physiologic systems. In the case where sensor systems collect data continuously and biomarkers are sampled serially or intermittently, these methods provide a way to integrate non-uniform datasets for other mathematical and statistical frameworks.

The nature of adding random error to the interpolated values means that for any iteration of this process, model performance will change—even if just slightly. To account for this within these analyses, we iterated through the random error generation step one hundred (100) times and subsequently analyzed model performance at each step—as outlined in Figure 2. This process creates a distribution that, theoretically, approaches the true value; however, the representation of variance and bias from each of these models can be observed in Figure 2 through the wider distributions and shifts in peaks, respectively. These distributions provide an example of the effect sizes between models for all subjects. Importantly, the small effect sizes (Cohen’s *d*) presented in Table 3 illustrate negligible differences between the total cortisol output calculated from the ground truth data compared to the interpolated data (from second- and third-degree polynomials), providing further evidence for the utility of these methods. Within Figure 2, an increase in model fit for the second- and third-degree polynomial regression models can be observed with corresponding reductions in model error (RMSE). While we do observe an increase in model fit and a corresponding reduction in model error for fourth-degree models and higher, these differences, relative to those changes observed between linear and second- and third-degree models, are not as great. While the optimal models were determined through careful evaluation of increases in model fit and reductions in model error, along with a desire for the most parsimonious model, an approximation of the effect sizes can be observed by how much these distributions overlap.

The performance of the models corresponding to the peak probabilities was stored and compared across individuals. Storing each iterative model is computationally inefficient so to regenerate the optimal model, we assigned a unique seed to each iteration. This process provides a simple and efficient way to implement these methods in practice. The randomness incorporated into the interpolation process outlined above provides error that is standardized to the observed data. This reduces the autocorrelation among the data compared to utilizing the interpolated data along the fitted line (interpolation without error). Many physiologic functions, including cortisol [36], display fractal and multifractal patterns [37]. The randomness incorporated into the methods presented here would not represent such dynamics but provides a framework for future work.

We believe that these methods show promise and have applications in diverse longitudinal datasets, particularly where biomarkers with drastically different half-lives (e.g., growth hormone vs. cortisol) are being collected. The specific sampling frequency necessary to address a research question should be determined by the investigators during the planning phase. A final methodological decision must be made after weighing all tradeoffs that may contribute to limitations and confounds of the work. However, this work provides an analytical framework for investigators to consider when making these decisions.

*Limitations*. In addition to its robust circadian and diurnal rhythms, cortisol is also responsive to acute and chronic stresses such as exercise and disease. This work has been limited to healthy adult males during rest and exercise conditions. In addition to the homogeneity of the current sample, it consists of a limited number of subjects. While there is substantial precedence for smaller sample sizes in studies with exceptionally high participant burden and where statistical power is generated through individual-level temporal data [1,13,14,24,38,39,40,41,42,43,44], the utilization and implementation of these methods necessitates validation in a larger, and more diverse, sample. Specifically, validation should be performed in females, across ages, and across the health spectrum. Sex differences in circadian cortisol output have been observed [45] and may be associated with the concentration of other circulating sex steroids [46] and confounded by hormonal contraceptive use [38]. Moreover, some have suggested sex differences in cortisol responses to stress tests [47,48], which, depending on the study design, may further influence optimal interpolation methodology. Further, age-related phase advance in cortisol circadian profiles [49] may dictate methodological considerations when working with older adults. Although beyond the scope of this analytic paper, it is known that diurnal cortisol is influenced by breast cancer [12] and diabetes [9], highlighting that varying degrees of overall health status should also be validated.

*Future directions*. This study utilized polynomial regression to fit the observed data and interpolate to a higher sampling frequency; however, other modeling techniques are frequently used to examine circadian trends. For example, cosinor-based analysis is also frequently used and may be particularly useful in similar data studies with extended data collection. These provide a robust method of rhythm detection and parameter estimation but sampling requirements, particularly for chronobiological analyses, can be difficult to meet [50]. Nevertheless, cosinor analysis can handle both equidistant and non-equidistant sampling frequencies and a similar approach outlined in this study should be explored in future analyses to determine and compare solutions [50].

With accessibility to previously unavailable datasets expanding due to open science, we can now leverage these prior works in secondary data analysis. Such work may aim to apply new mathematical and statistical methodologies to better understand the interconnectedness between physiologic systems. For example, dynamic P-technique is a structural equation model used to examine the temporal relations between constructs in a single, or multiple, individual(s) across time. These models provide significant flexibility and the ability to quantify specific relations and effects, including time lags, time dependencies, and equivalent relations across the sample. From a predictive analytics framework, recurrent neural networks and long-short-term memory neural networks provide a framework to either predict an outcome feature at the same timestep or forecast those values into the future. Whether within the structural equation modeling framework or the use of neural networks, equal observations between each of the features are helpful, if not necessary. In cases where one feature/biomarker has inherently different trends (e.g., longer/shorter half-life) and measurement at higher frequencies is cost- and time-prohibitive, the present analyses provide a framework for interpolating these data to reach the necessary sampling frequency.

## 5. Conclusions

This study examined how various sampling methodologies and methods of interpolation impact estimations of 24 h cortisol output and established a data-driven approach to address issues of noncurrent biological sampling within research designs. We conclude that third-degree and second-degree polynomial regressions can be used to fit a variety of serum and salivary cortisol sampling methods, respectively, and used to interpolate to higher sampling frequencies without compromise. These methods have applications for biomarker research as well as diverse longitudinal datasets where, for example, hormones with different half-lives (e.g., growth hormone) and/or sensor systems and serum or salivary cortisol data are collected at different sampling frequencies. Future work should be performed to validate these findings in more diverse samples, as well as other biomarkers.

## Figures and Tables

**Table 1:** Outline of the sampling methods that were tested for serum and salivary cortisol. Polynomial validation from degree 1 through various max degrees was fit to each profile. Serum cortisol was analyzed for all the following sampling methods with Q60 (sample taken every 60 min) serving as the ground truth data for serum. Salivary cortisol was analyzed for Q120, Q240, Q360, INT1, and INT2 with Q120 (sample taken every 120 min) serving as the ground truth data for saliva.

Time (h)	Q60	Q120	Q180	Q240	Q360	INT1	INT2
0	X	X	X	X	X	X	X
1	X						
2	X	X				X	X
3	X		X				
4	X	X		X		X	
5	X						
6	X	X	X		X		X
7	X						
8	X	X		X		X	
9	X		X				
10	X	X					
11	X						
12	X	X	X	X	X		
13	X						
14	X	X				X	
15	X		X				
16	X	X		X			X
17	X						
18	X	X	X		X		
19	X						
20	X	X		X			
21	X		X				
22	X	X					
23	X						
24	X	X	X	X	X	X	X
Max Degree	6	6	5	4	3	3	3

**Table 2:** Estimated 24 h serum and salivary cortisol output (μg/dl) for ground truth (i.e., sampled every 60 min(Q60) and 120 min (Q120) for serum and salivary cortisol, respectively), observed data for each alternative sampling method, and following interpolation from polynomial degrees 2–3.

	Condition	Timing	Obs.	Degree 2	Degree 3
	rest	Q60	11,135.85 (2300.29)		
Serum	exercise	Q60	10,024.43 (1030.94)		
	rest	Q120	11,065.8 (2280.6)	11,077.09 (2204.25)	11,046.32 (2178.71)
	exercise	Q120	9865.48 (1033.59)	10,057.14 (1073.98)	10,070.29 (1057.1)
	rest	Q180	11,191.01 (2203.76)	11,118.78 (2140.97)	11,334.01 (2263.54)
	exercise	Q180	9985.59 (967.02)	10,176.84 (996.33)	10,152.51 (1036.76)
	rest	Q240	11,018.2 (2041.66)	11,195.07 (2344.68)	1,1206.2 (2205.18)
	exercise	Q240	10,102.93 (1091.82)	10,231.22 (1075.46)	10,139.43 (1065.69)
	rest	Q360	11,468.69 (2101.58)	11,466.19 (2374.9)	11,309.24 (2329.93)
	exercise	Q360	10,208.27 (952.14)	10,277.51 (1029.56)	10,131.38 (1035.5)
	rest	INT1	12,265.24 (2399.45)	11,880.72 (2359.11)	11,665.48 (2308.37)
	exercise	INT1	12,265.24 (2399.45)	10,442.72 (1101.73)	10,438.46 (1083.53)
	rest	INT2	11,201.09 (2318.1)	11,489.27 (2351.78)	11,531.46 (2314.36)
	exercise	INT2	11,201.09 (2318.1)	10,398.87 (1077.02)	10,442.51 (1044.94)
	rest	Q120	641.34 (112.96)		
Saliva	exercise	Q120	508.51 (80.64)		
	rest	Q240	644.44 (118.56)	657.33 (114.49)	657.09 (114.64)
	exercise	Q240	533.71 (78.89)	538.97 (80.52)	538.79 (80.04)
	rest	Q360	727.16 (115.97)	689.99 (110.93)	688.8 (111.03)
	exercise	Q360	584.21 (95.73)	555.84 (85.08)	556.47 (85.24)
	rest	INT1	634.65 (100.82)	689.06 (113.59)	687.46 (112.95)
	exercise	INT1	525.09 (80.06)	554.17 (86.13)	553.92 (85.88)
	rest	INT2	618.25 (115.07)	687.74 (116.57)	688.78 (116.03)
	exercise	INT2	515.87 (78.11)	558.39 (81.61)	558.05 (81.6)

Data are presented as mean (SE). Mean estimates as well as lower and upper bounds of the confidence interval from the two one-sided tests for equivalence are provided in Table 3.

**Table 3:** Mean estimates, confidence intervals, and effect sizes from the two one-sided tests for equivalence (TOST).

					Degree 2				Degree 3	
	Condition	Timing	*d*	Estimate	Lower	Upper	*d*	Estimate	Lower	Upper
Serum	rest	Q120	0.001	−0.007	−0.008	−0.005	0.001	−0.007	−0.008	−0.006
	exercise	Q120	0.001	−0.001	−0.002	0.000	0.001	−0.001	−0.002	0.000
	rest	Q180	0.001	−0.015	−0.017	−0.013	0.002	−0.015	−0.017	−0.013
	exercise	Q180	0.002	−0.012	−0.014	−0.010	0.002	−0.012	−0.014	−0.011
	rest	Q240	0.001	−0.018	−0.020	−0.016	0.001	−0.018	−0.020	−0.016
	exercise	Q240	0.003	−0.017	−0.020	−0.015	0.002	−0.017	−0.019	−0.015
	rest	Q360	0.004	−0.023	−0.025	−0.020	0.002	−0.023	−0.025	−0.022
	exercise	Q360	0.004	−0.019	−0.022	−0.017	0.002	−0.020	−0.022	−0.018
	rest	INT1	0.008	−0.055	−0.058	−0.053	0.006	−0.056	−0.057	−0.054
	exercise	INT1	0.006	−0.045	−0.046	−0.043	0.006	−0.045	−0.046	−0.043
	rest	INT2	0.004	−0.037	−0.039	−0.035	0.005	−0.037	−0.038	−0.036
	exercise	INT2	0.005	−0.041	−0.043	−0.039	0.006	−0.041	−0.042	−0.040
Saliva	rest	Q240	0.005	−0.029	−0.033	−0.025	0.005	−0.030	−0.033	−0.026
	exercise	Q240	0.013	−0.061	−0.066	−0.056	0.013	−0.061	−0.066	−0.056
	rest	Q360	0.015	−0.080	−0.087	−0.074	0.015	−0.082	−0.088	−0.077
	exercise	Q360	0.020	−0.090	−0.095	−0.085	0.020	−0.092	−0.096	−0.087
	rest	INT1	0.015	−0.084	−0.091	−0.077	0.015	−0.084	−0.091	−0.078
	exercise	INT1	0.019	−0.084	−0.091	−0.077	0.019	−0.085	−0.092	−0.079
	rest	INT2	0.015	−0.079	−0.084	−0.074	0.015	−0.079	−0.084	−0.075
	exercise	INT2	0.021	−0.096	−0.103	−0.089	0.021	−0.098	−0.104	−0.092

Estimates are the mean difference between log-transformed estimates of total cortisol output from observed values and those from interpolated values with corresponding lower and upper values of the confidence interval. TOST tests suggested statistical similarity between all presented comparisons (*p* < 0.05). The small effect sizes (Cohen’s *d*) presented here represent negligible differences between the total cortisol output calculated from the ground truth data compared to the interpolated data (from second- and third-degree polynomials).

## Data Availability

The data that support the findings of this study are available upon reasonable request from the authors.
